# Unusual Presentation of Metastatic Medullary Thyroid Cancer Involving Bone Marrow, Kidneys, and Adrenal Gland: A Literature Review Based on a Case Report

**DOI:** 10.1002/cnr2.70022

**Published:** 2024-10-16

**Authors:** Pouya Ebrahimi, Moloud Payab, Alireza Shariati, Neda Alipour, Aysan Nozheh, Seyed Mohammad Tavangar, Homa Taheri, Mahbube Ebrahimpur

**Affiliations:** ^1^ Tehran Heart Center, Cardiovascular Disease Research Institute Tehran University of Medical Sciences Tehran Iran; ^2^ Non‐Communicable Diseases Research Center, Endocrinology and Metabolism Population Sciences Institute Tehran University of Medical Sciences Tehran Iran; ^3^ Department of Internal Medicine, Shariati Hospital, School of Medicine Tehran University of Medical Sciences Tehran Iran; ^4^ Pathology Resident, Shariati Hospital, School of Medicine Tehran University of Medical Sciences Tehran Iran; ^5^ Department of Pathology, Dr. Shariati Hospital Tehran University of Medical Sciences Tehran Iran; ^6^ Chronic Diseases Research Center, Endocrinology and Metabolism Population Sciences Institute Tehran University of Medical Sciences Tehran Iran; ^7^ Cedars‐Sinai Cardiology Department Los Angeles California USA; ^8^ Endocrinology and Metabolism Research Center, Endocrinology and Metabolism Clinical Sciences Institute Tehran University of Medical Sciences Tehran Iran; ^9^ Elderly Health Research Center, Endocrinology and Metabolism Population Sciences Institute Tehran University of Medical Sciences Tehran Iran

**Keywords:** adrenal gland, bone marrow, case report, COVID‐19, medullary thyroid cancer, metastasis

## Abstract

**Background:**

Medullary thyroid cancer (MTC) is one of the rare neuroendocrine malignancies. This cancer is hereditary in approximately 20% of cases. Although lymph node (LN) metastasis is prevalent in MTC, distant metastasis is not commonly seen in these patients. The most common locations for metastasis are the lungs, liver, and bones. This study presents an extremely rare MTC metastasis to bone marrow (BM) and adrenal gland, which has not been reported before.

**Case:**

The patient was a 50‐year‐old man with a diagnosis of MTC and total thyroidectomy 2 months before his presentation. He came to the emergency department (ED) complaining of dyspnea, diffuse bone pain, nonbloody diarrhea, and abdominal cramps starting in the last month before. Initial treatment with intravenous fluid infusion and loperamide, due to the provisional diagnosis of infectious diarrhea, was ineffective. Further assessments revealed severe pancytopenia and a massive tumor above the left kidney. Bone marrow aspiration (BMA) and biopsy (BMB) led to the diagnosis of invasive metastasis of the MTC to the BM and the left adrenal gland. In the initial evaluations, his COVID‐19 test became positive, and despite all efforts, his condition deteriorated, and he died 5 days after admission due to respiratory distress.

**Conclusion:**

Most MTC cases present with thyroid nodules in the initial steps and are confined to the thyroid gland or the adjacent LNs. These cases are mostly cured by thyroidectomy and LN dissection. This neuroendocrine cancer infrequently becomes aggressive and involves other parts of the body. However, involving BM or adrenal gland has been scarcely reported. Due to ineffective red and white blood cell production, BM metastasis can cause pancytopenia and, consequently, pallor, fatigue, dyspnea, and susceptibility to infections. High calcitonin levels can also cause diarrhea. The initial diagnosis is mostly with neck ultrasound (US) and fine needle aspiration (FNA). Total thyroidectomy is the main therapeutic option for these patients. Calcitonin and carcinoembryonic antigen (CEA) are sensitive indicators of recurrence or remaining tumors, which might be helpful for the initial diagnosis and postoperation follow‐up. Although extremely rare, invasive metastasis of MTC might involve unusual body organs such as the BM or adrenal glands. In cases of unjustifiable pancytopenia or adrenal dysfunction in MTC‐positive patients, these possibilities should be considered and ruled out by some specific evaluations, such as bone marrow biopsy and contrast‐enhanced imaging.

**Abbreviations**
BMA: bone marrow aspiration
BMB: bone marrow biopsy
CEA: carcinoembryonic antigen
CT: computed tomography
EBRT: external beam radiotherapy
FNA: fine needle aspiration
GI: gastrointestinal
IV: intravenous
MEN: multiple endocrine neoplasia
MRI: magnetic resonance imaging
MTC: medullary thyroid carcinoma
RCT: randomized clinical trial
SAD: shortest axial diameter
TKI: tyrosine kinase inhibitor

## 1 Background

Although most mortalities and morbidities caused by cancers are seen in more prevalent malignancies such as breast, prostate, lung, and colon cancers, less common ones can also present as aggressive and lethal malignancies, such as medullary thyroid cancers (MTC), which have been diagnosed with distant metastasis [1, 2, 3]. MTC, which originates from the neuroendocrine (parafollicular) cells in the upper part of the thyroid lobes, is considered a rare malignancy, being diagnosed in only 5% of total thyroid cancers and 13% of thyroid‐cancer‐induced deaths [3, 4] It has been shown that the incidence of MTC has increased from 0.41/100 000 between 1986 and 1996 to 0.57/100 000 between 2008 and 2018. This increased incidence might be caused by the improvement in diagnostic modalities, such as ultrasonography of the thyroid [3]. Approximately 75%–80% of MTC cases are sporadic, primarily diagnosed in the fifth and sixth decades of life. In contrast, 20%–25% are cases of hereditary subtypes, including multiple endocrine neoplasia (MEN2) and familial MTC (FMTC)‐related cases that are primarily presented in the second or the third decades of life [3, 5]. Considering that RET gene mutations have been detected in most cases of hereditary MTC, these types of MTC, including FMTC and MEN2‐positive cases, can be considered RET gene mutation‐related cancers [5]. Approximately 15%–20% of MTC cases have distant metastasis when the malignancy is diagnosed [6]. Moreover, it has been shown that patients with distant metastasis have a poorer prognosis (5‐year survival of 38%) compared to the localized cases of MTC patients (5‐year survival of more than 90%) [3].

MTC presentation consists of a solitary thyroid nodule US detected in 75%–95% of patients. However, calcitonin screening reduced the presentation of locally advanced or metastatic patients. More than two‐thirds of cases have lymph node (LN) involvement at the time of diagnosis, and the large size of the tumor might lead to pressure symptoms, including dyspnea, hoarseness, and dysphagia. However, calcitonin screening reduced the presentation of locally advanced or metastatic patients [7, 8, 9]. Lungs, liver, bones, brain, and soft tissues are more commonly involved organs. In contrast, the involvement of other organs, such as the kidney and adrenal glands, has rarely been reported before. The long‐distance spread of cancerous cells in this malignancy mainly occurs through blood [7, 8].

This study presents a case of MTC cancer with multiple unusual and rarely reported metastases that came to the emergency department due to dyspnea.

## 2 Case

A 50‐year‐old male presented to the emergency department of Shariati Hospital of Tehran (October 22, 2023) complaining of nonbloody diarrhea, abdominal cramps, and generalized bone pain, which had begun a month before his presentation. He also mentioned dyspnea, nonproductive cough, sharp chest pain, painful swallowing, and losing about 10 kg during the last month. The patient was a previous smoker without any remarkable familial history. In his past medical history, he had undergone a total thyroidectomy 6 months before with the initial diagnosis of papillary thyroid cancer. However, after the total thyroidectomy, central and lateral LN dissection, and removal of a part of the tumor, which had invaded the mediastinum and microscopic evaluation, the diagnosis of MTC was made. Despite being recommended by the medical team, further assessment for metastasis was not made due to the patient's lack of consent. Moreover, the patient had ignored his follow‐up sessions and had requested to withdraw therapeutic medications after the surgery. No further details were documented regarding his initial admission and surgery. On physical examination, he was tachypneic (respiratory rate: 30/min), had low blood pressure (90/60 mmHg), and his oxygen saturation level (SpO_2_) without an oxygen mask was 79%. Skin and conjunctival pallor, generalized bilateral crackle in lungs, using abdominal muscles for breathing, mild bilateral nonpitting lower extremity edema, and a palpable right parasternal mass on the surgical scar were also detected.

**FIGURE 1:**
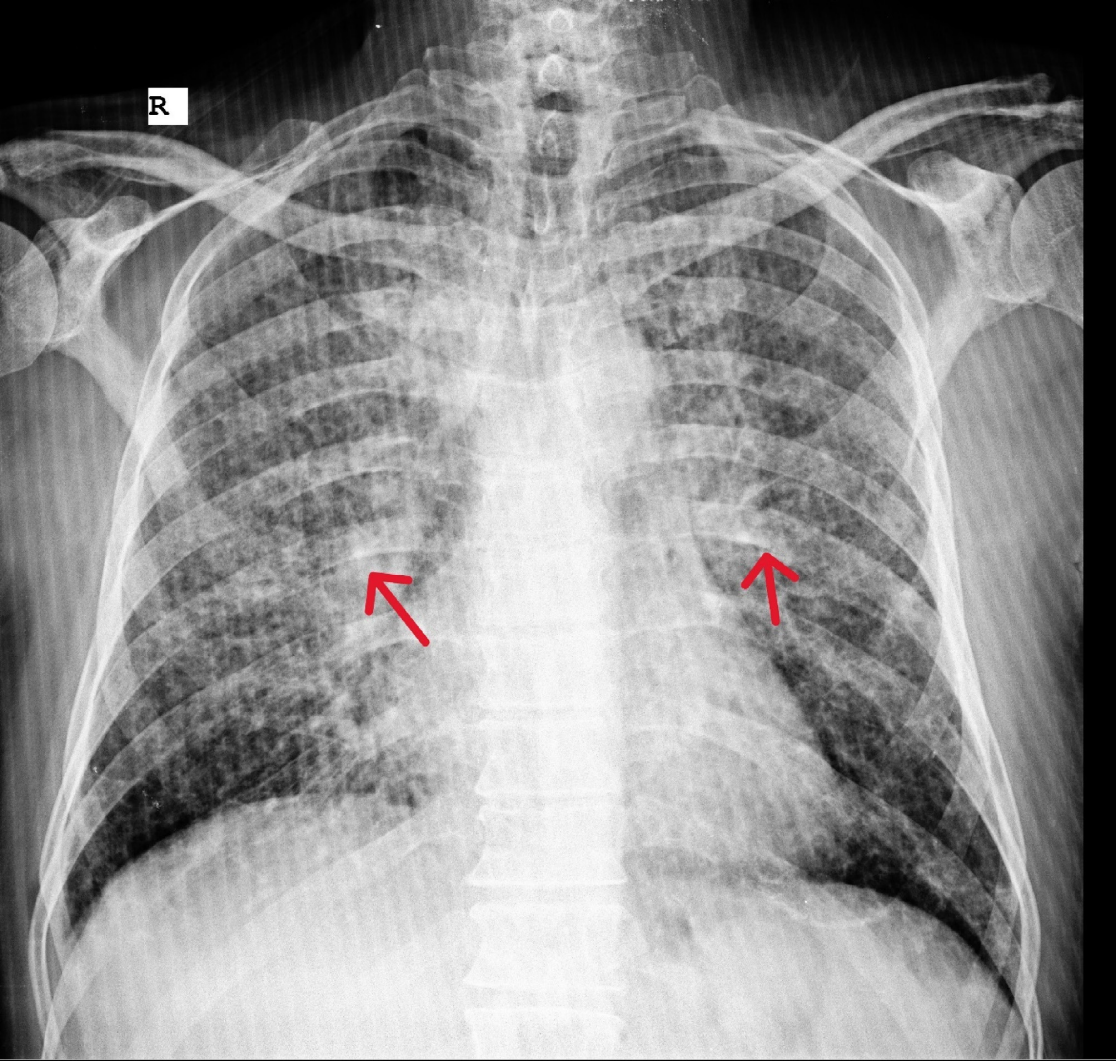
Bilateral generalized nodular pattern and ground glass opacity appearance in chest x‐rays of the patients are suggestive of a coincidence of COVID‐19 infection and metastasis of cancer to both lungs (red arrows: nodular pattern and ground glass opacity appearance).

**FIGURE 2:**
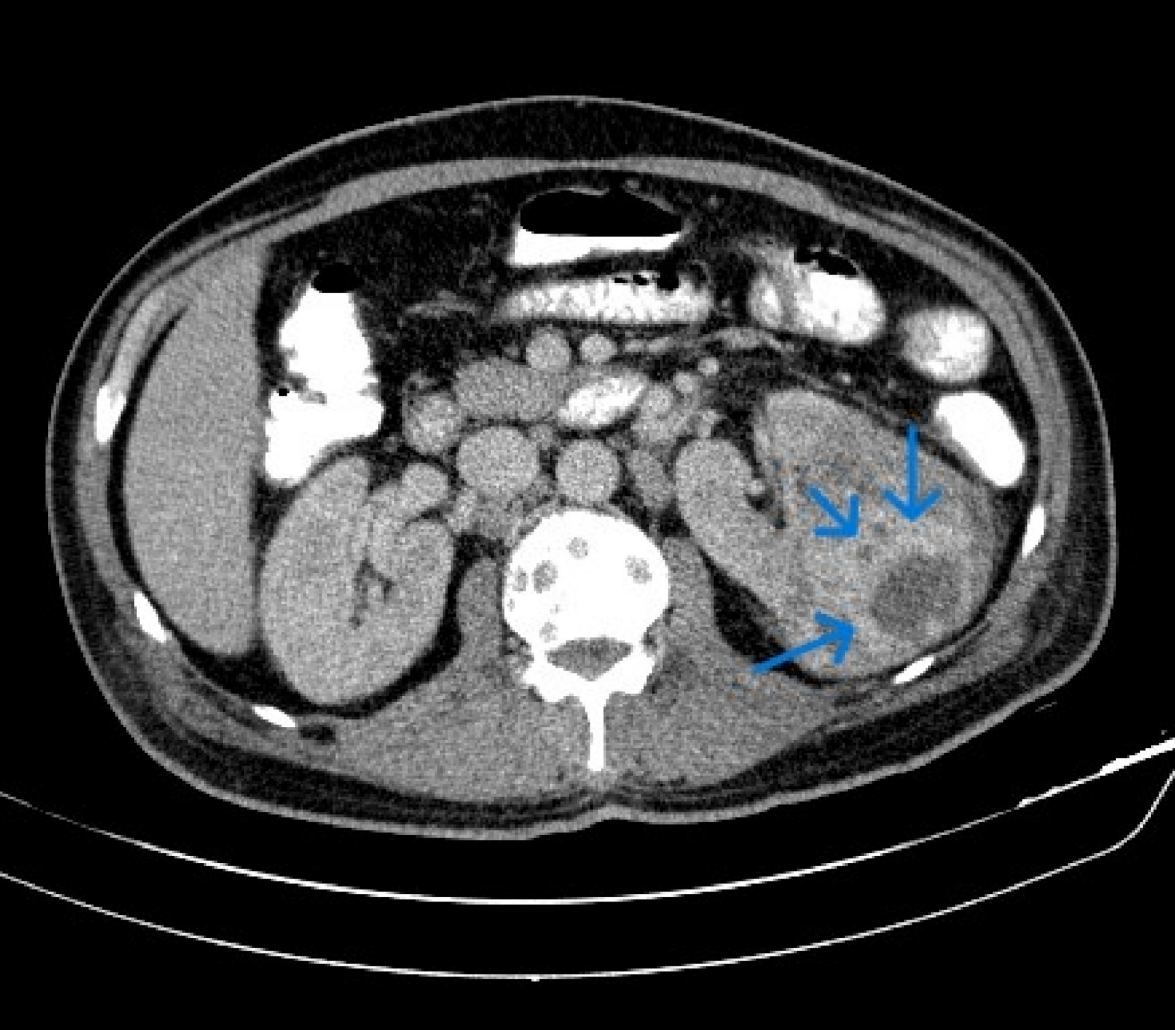
Abdominal (CT) scan with and without oral and IV contrast showed large heterogeneous enhancing lesions at the middle portion of the left kidney (blue arrow), which was suggestive of MTC metastasis to the kidney and the adrenal gland.

The patient was admitted with a provisional diagnosis of infectious diarrhea. Initial stool tests for *Clostridium difficile* were performed, and the treatment started with intravenous (IV) fluid, which had no remarkable effect. Stool tests ruled out infectious diarrhea. The IV fluid treatment and loperamide were also started, which caused significant improvement in the patient's condition. After having the results of his laboratory exams (Table 1), fresh frozen plasma (FFP) infusion was also initiated to improve his pancytopenia, which did not cause any remarkable improvement in his platelet count. A chest x‐ray was performed, which showed a bilateral generalized nodular pattern, and the ground glass opacity appearance was suggestive of a coincidence of COVID‐19 infection and metastasis of cancer (Figure 1). A thyroid bed ultrasonography was performed, which revealed no significant mass but a few LNs (maximal SAD of 7.5 mm) in the right second zone of the neck (Table 2). The abdominal US revealed an isoechoic mass‐like lesion in the upper pole of the left kidney. An abdominopelvic computed tomography (CT) scan with and without oral and IV contrast after the preparation of the patient was done, which showed large (77 × 58 × 54 mm) heterogeneous enhancing lesions at the middle portion of the left kidney with multiple adjacent and para‐aortic lymphadenopathies up to 15 mm, a 25 × 20 mm lesion at the lateral limb of the left adrenal gland (Figure 2), two small hypo‐dense lesions (7–8 mm) at Segment V‐VI of the liver, and diffuse lytic bone lesions, all of which were suspicious of metastatic involvement. In the patient's laboratory test, remarkably elevated calcitonin and carcinoembryonic antigen (CEA) were also hints for the recurrence of MTC. Therefore, a biopsy of the detected tumor at the upper pole of the left kidney under the guidance of the CT scan was planned. Also, due to the detection of resistant pancytopenia, bone marrow aspiration and analysis were performed.

**TABLE 1:** Laboratory tests on the second day of his admission.

Laboratory indicator	Serum level	Normal range
Hemoglobin	10.5 g/dL	13.1–17.2 g/dL
Peripheral blood smear	There is anisocytosis and polychromasia	
Mean corpuscular volume	85 fL	82–90 fL
Reticulocyte count	3.8%	< 0.1
Calcium	13.5 mg/dL	8.5–10.5 mg/dL
Phosphorus	5.2 mg/dL	2.5–5.5 mg/dL
Parathyroid hormone	< 3 pg/mL	11–67 pg/mL
Thyroid stimulating hormone	13 mIU/L	0.32–3.20
Serum thyroglobulin	0.2 IU/L	< 10 IU/L
Serum antithyroglobulin	1 IU/L	< 50 IU/L
Serum calcitonin	810 pg/mL	< 8.4 pg/mL
CEA	669 ng/mL	< 5 ng/mL
24‐h urine metanephrine	640 μg	< 600 μg
24‐h urine vanillylmandelic	19 mg	≤ 13.6 mg
Ferritin	665 ng/mL	12–300 ng/mL
Erythrocyte sedimentation rate	Elevated	Normal
C‐Reactive protein	Elevated	Normal
Fibrin degradation products	Elevated	Normal
D‐Dimer	Elevated	Normal
Lactate dehydrogenase	1368 units/L	105–233 units/L

**TABLE 2:** Results of the patient's radiologic evaluation.

Type of radiologic modality	The result
Chest x‐ray	The bilateral generalized nodular pattern and ground glass opacity appearance were suggestive of a coincidence of COVID‐19 infection and metastasis of cancer.
Thyroid US	No significant mass was revealed, but there were a few lymph nodes (maximal SAD of 7.5 mm) in the right second zone of the neck.
Abdominal US	Demonstrated an isoechoic mass‐like lesion in the upper pole of the left kidney.
Chest CT scan	Multiple pulmonary nodules with interlobular septal thickening suggestive of metastases and lymphangitic carcinomatosis were evident. Also, a 74 × 37 mm soft tissue mass in the upper anterior chest wall associated with the destruction of the sternal manubrium, multiple mediastinal, and bihilar LAPs, multiple lytic lesions in favor of metastasis, and a 19 × 22 mm nodule in the left adrenal were detected.
Abdominal CT scan ± IV and oral contrast	It showed a large (77 × 58 × 54 mm) heterogeneous enhancing lesion at the middle portion of the left kidney with multiple adjacent and para‐aortic lymphadenopathies up to 15 mm, a 25 × 20 mm lesion at the lateral limb of the left adrenal gland, two small hypo‐dense lesions (7–8 mm) at Segment V–VI of the liver, and diffuse lytic bone lesions.
Bone scan (TC‐99 diphosphate)	A bone scan revealed significant uptake in the right aspect of the sternal body, mid‐shaft of the right humerus, and the intertrochanteric region of the left femur.
Pet scan (Ga‐68‐DOTATATE)	There are demonstrated metastatic lesions in almost all axial and appendicular bones, several bilateral pulmonary metastases, several mediastinal and a few para‐aortic and aorto‐caval lymph nodes, a large exophytic mass originating from the left kidney (as seen in the abdominal CT scan), and a left adrenal nodule.

**FIGURE 3:**
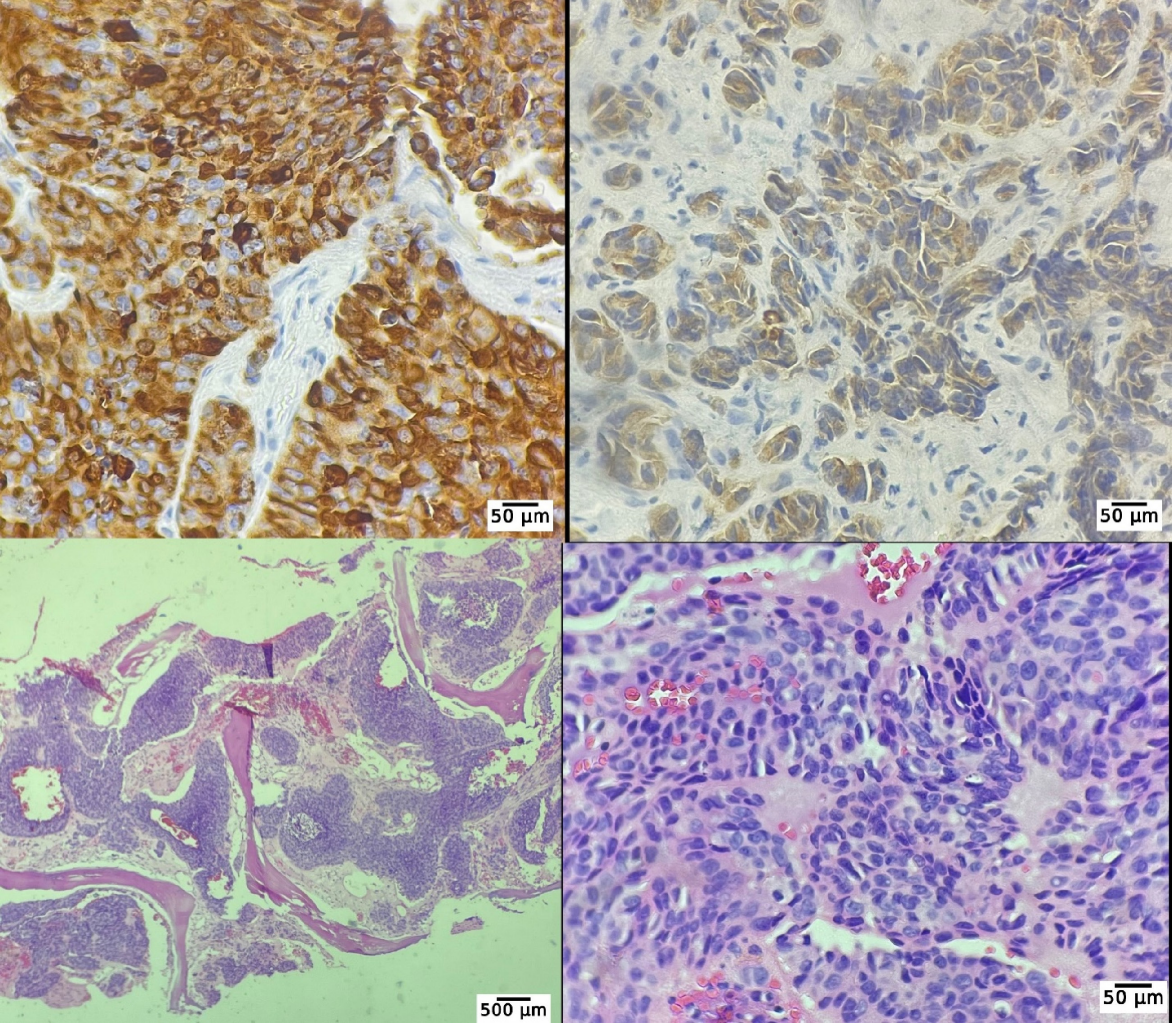
Bone marrow biopsy pathology evaluation showed nests of tumoral cells composed of round to oval nuclei, inconspicuous nucleoli, and a moderate amount of eosinophilic cytoplasm. Left up: chromogranin ×400; right up: synaptophysin ×400; left down: hematoxylin and eosin ×40, marrow spaces composed of nests of nonhematopoietic cells; right down: hematoxylin and eosin ×400, nest of tumor cells are seen in this image.

**FIGURE 4:**
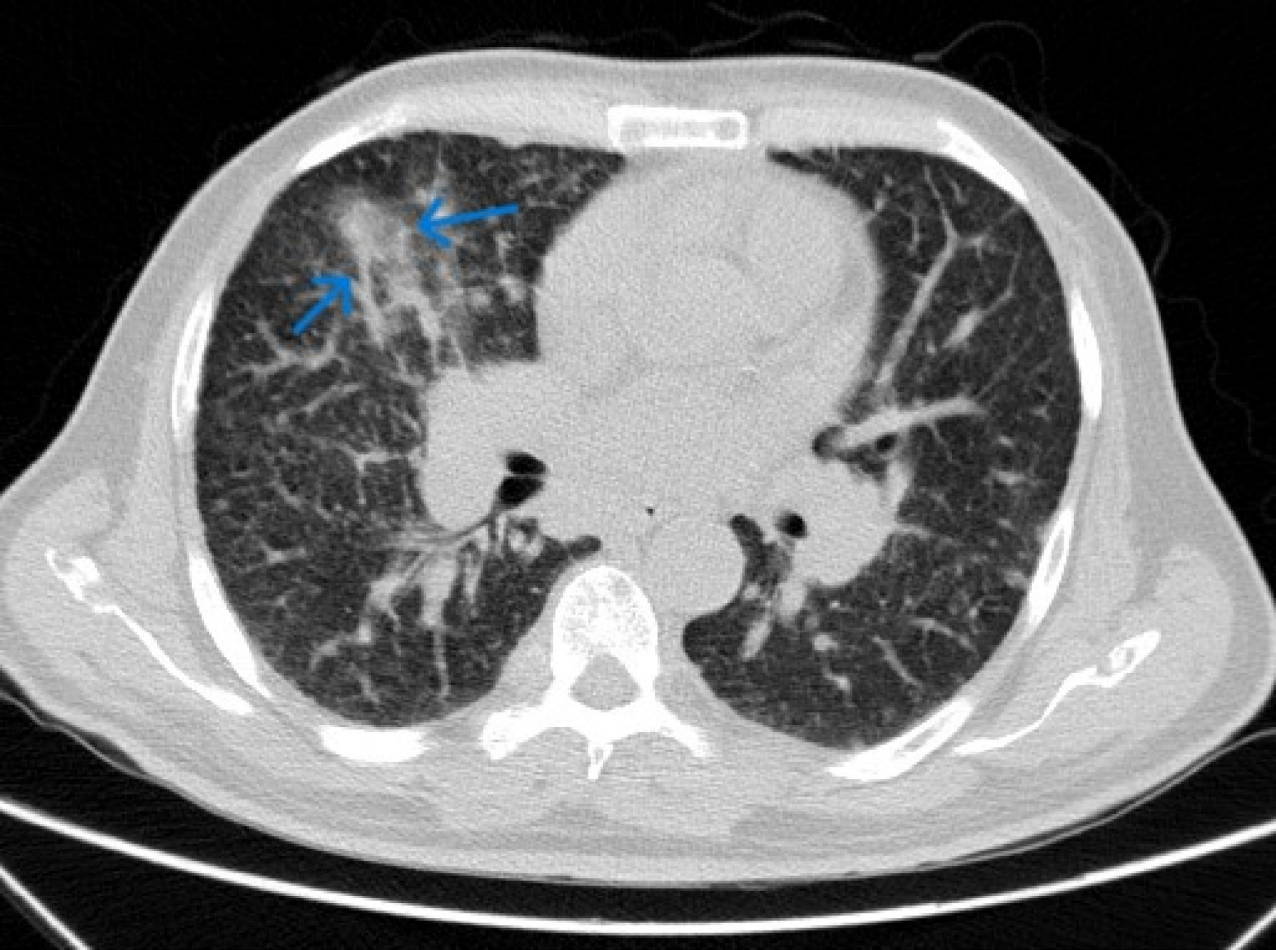
Spiral chest CT scan showed multiple pulmonary nodules with interlobular septal thickening (blue arrow) suggestive of metastases and lymphangitic carcinomatosis.

**FIGURE 5:**
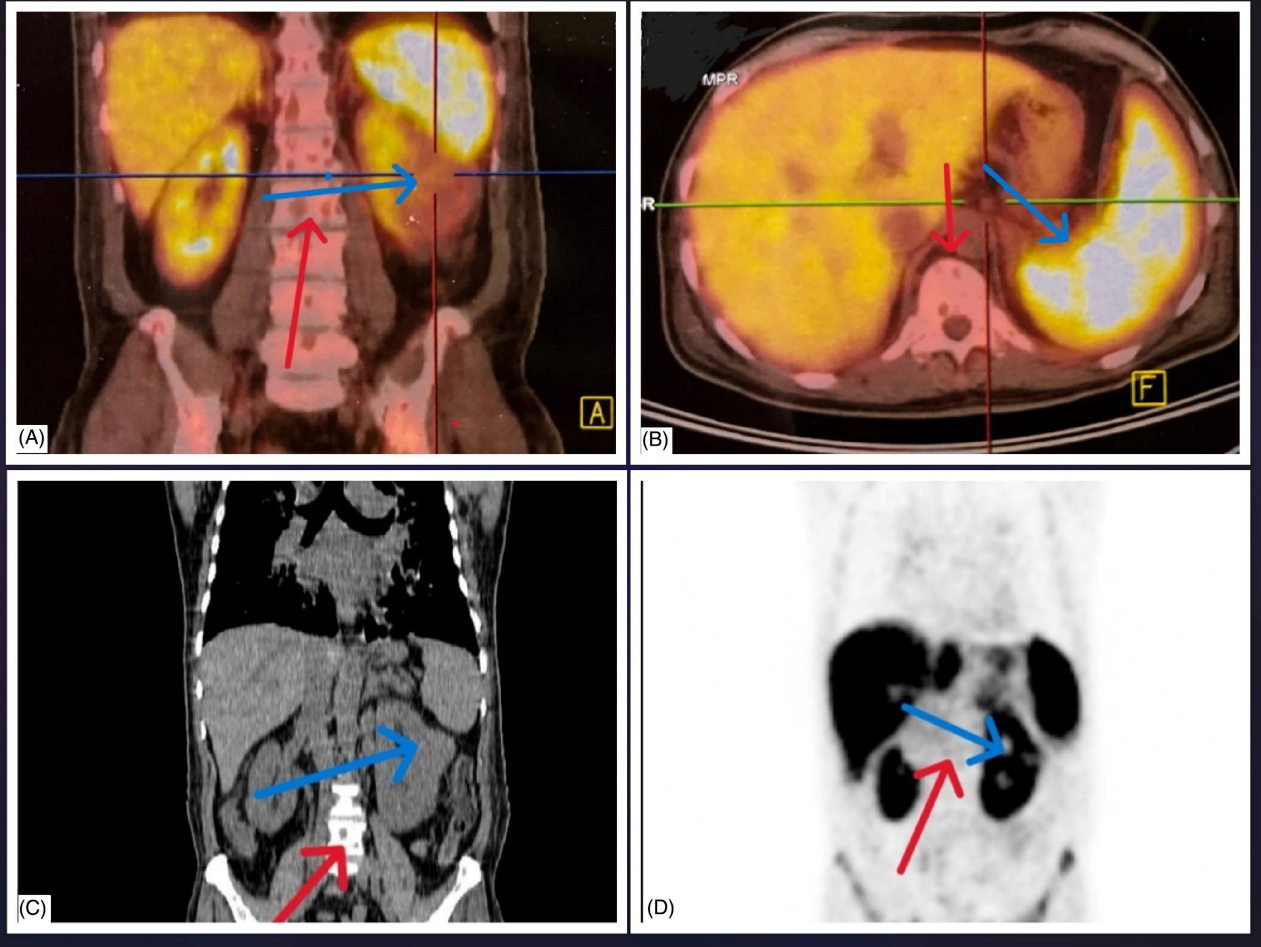
PET scan showed metastatic lesions in almost all axial (red arrow) and appendicular bones. A large exophytic mass originates from the left kidney (blue arrow). (A) Fusion‐Mode, Coronal Plane, (B) Fusion‐Mode, Axial Plane, (C) PET CT‐Mode, Coronal Plane, (D) PET Scan, Coronal Plane.

**FIGURE 6:**
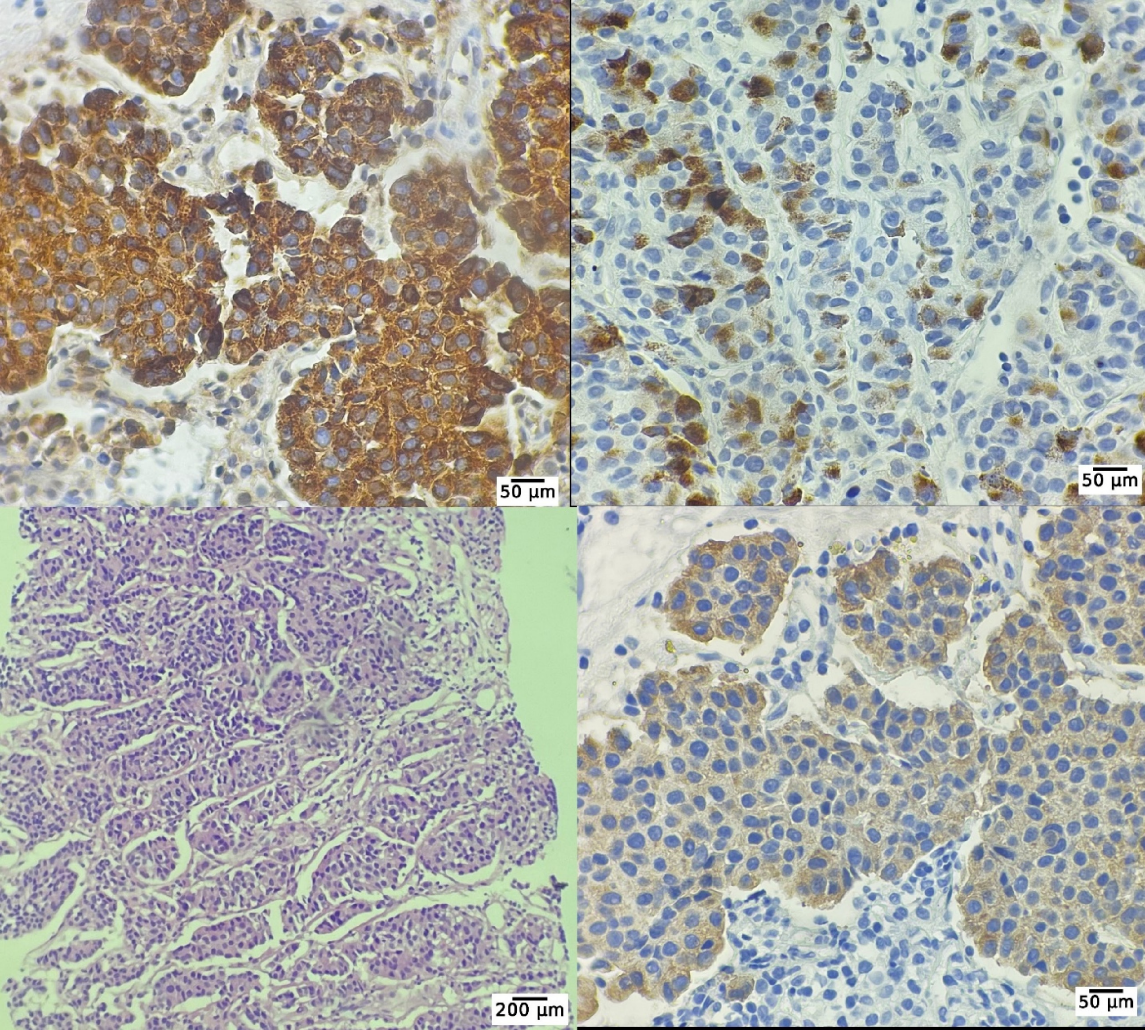
Renal mass biopsy containing delineated and infiltrative tumors containing cells without cohesion. Left up: chromogranin ×400, showing poorly differentiated cancerous cells infiltrating the kidney; right up: calcitonin ×400, positive immunocytochemical staining for calcitonin is suggestive of MTC; left down: hematoxylin and eosin ×100, nests of uniform tumoral cells are seen; right down: synaptophysin ×400.

Involvement of the bone marrow due to MTC metastasis was confirmed after a bone marrow biopsy. The immunohistochemistry revealed that the bone marrow biopsy was positive for synaptophysin, chromogranin, calcitonin, and Ki67 index ≥ 5% (Figure 3). Considering a very high level of calcitonin (810 pg/mL) and a history of thyroidectomy, the diagnosis of invasive MTC was at the top of the list. Moreover, the elevated CEA (665 ng/mL) made the metastatic and aggressive MTC the most probable differential diagnosis. Regarding respiratory symptoms and fever, a swab test from the nasopharynx and PCR for COVID‐19 result was positive. With the confirmed diagnosis of the MTC recurrence, evaluation for more metastatic invasions started. A spiral chest CT scan was performed that revealed multiple pulmonary nodules with interlobular septal thickening suggestive of metastases and lymphangitic carcinomatosis were evident. Also, a 74 × 37 mm soft tissue mass in the upper anterior chest wall associated with the destruction of the sternal manubrium, multiple mediastinal and bihilar LAPs, multiple lytic lesions in favor of metastasis, and a 19 × 22 mm nodule in the left adrenal were detected (Figure 4). Color Doppler US of the lower limbs and echocardiogram were unremarkable. Also, a bone scan revealed significant uptake in the right aspect of the sternal body, mid‐shaft of the right humerus, and the intertrochanteric region of the left femur, all suggesting malignancy. Moreover, a PET/CT scan using Ga‐68‐DOTATATE demonstrated metastatic lesions in almost all axial and appendicular bones, several bilateral pulmonary metastases, several mediastinal and a few para‐aortic and aortocaval LNs, a large exophytic mass originating from the left kidney (as seen in the abdominal CT scan), and a left adrenal nodule (Figure 5). The left renal mass was sampled using a core needle biopsy. The histological and IHC findings were compatible with those of high‐grade MTC metastasis. The IHC revealed that the renal mass was positive for synaptophysin, chromogranin, calcitonin, and Ki67 index ≥ 5%, as was the bone marrow sample (Figure 6).

Following discussing the diagnosis and therapeutic options with the patient and his family and aligned with their decision to initiate palliative therapy, the treatment continued with supportive methods, and he was transferred to the Intensive Care Unit (ICU). Five days after admission, the patient's condition deteriorated, his respiratory distress got worse, and his oxygen saturation declined continuously. Finally, despite all efforts and therapeutic procedures, the patient had a cardiac arrest on the fifth day of his admission, and he died.

## 3 Conclusion

This study presents a case of MTC with multiple metastatic lesions to the kidney, adrenal glands, bone marrow, and lungs. Therefore, this type of cancer can be a lethal and aggressive malignancy, which mostly involves the skeleton, liver, brain, and lungs in cases of long‐distance metastasis [10, 11]. In conclusion, early diagnosis and appropriate staging are crucial for planning an effective treatment strategy [12]. Distant metastasis can less commonly involve the pleura, heart, ovary, pancreas, pituitary, retina, skin, and breast, as seen in the present study, and rarely involve adrenal glands and bone marrow [13, 14]. To the authors' knowledge, only one MTC metastasis to the BM has been reported so far [15], and this study is the first report of simultaneous metastasis of MTC to bone marrow, adrenal, and kidney.

It is important to note that a calcitonin level of > 100 pg/mL in a patient with a thyroid nodule (or the history of thyroidectomy such as the presented case in this study) has been shown to be consistent with the diagnosis of MTC. Moreover, the higher level of calcitonin is associated with an increased risk of metastasis and more invasive MTC [16]. Furthermore, CEA is associated with a more aggressive type of MTC [17]. CEA doubling time is considered a good way to predict prognosis and classify patients into stable and progressive disease subgroups [18]. An elevated level of other biomarkers, such as an elevated neutrophil‐to‐lymphocyte ratio (NLR), platelet‐to‐lymphocyte ratio (PLR), and systemic immune‐inflammatory index (SII), has been considered a negative prognostic factor in several neoplasms. However, the association between them and MTC has not been clearly stated [19].

American Thyroid Association guidelines for Managing MTC (2015) [9] recommends that after FNA confirms MTC, LN metastasis should be examined by neck US. They also suggest performing (1) a contrast‐enhanced CT scan of the neck and chest, (2) a three‐phase contrast‐enhanced liver protocol CT or MRI of the liver, (3) a bone scan and axial MRI for patients with serum calcitonin levels > 500 pg/mL or other signs of distant metastasis [9]. However, in some cases, these imaging modalities are not diagnostic, and unusual evaluations are needed for the detection of cancer and metastasis, such as our patient, who was diagnosed with metastasis to bone marrow with BMA (Table 3) [15]. Unfortunately, these diagnostic features were not performed in our case to avoid the patient's further workup after the thyroidectomy.

**TABLE 3:** Unusual metastatic cases of MTC literature review.

Article/authors	Age and gender	Presentation	Treatment/progression or remission of the disease
Aggressive bone marrow metastatic medullary thyroid carcinoma/Lesesve J. et al. [15]	48‐year‐old woman	Presented with cervical pain/a palpable thyroid nodule was found surrounded by enlarged lymph nodes/a high serum level of calcitonin and low calcium/elevated CEA/Histopathological examination demonstrated sheets of polygonal cells with the pseudo glandular arrangement and angioinvasion, 1 year after the surgery due to pancytopenia BMA was done and showed total infiltration composed of clumps of round to spindle cells separated by amorphous deposits positive tumor cells for calcitonin, chromogranin A and synaptophysin.	Extensive thyroidectomy and LN dissection were performed. However, the disease progressed and metastasized to the liver/and then BM involvement was diagnosed by immunohistochemistry, confirming the participation of parafollicular calcitonin‐producing C‐cells. Calcitonin and CEA levels were elevated. The patient died soon despite being treated with chemotherapy.
Metastatic medullary thyroid cancer to the brain: A case report and review of the literature/Sastry R. et al. [20]	53‐year‐old male	Firstly, diagnosed with MTC in 1995/presented with a large right thyroid nodule/elevated calcitonin and then thyroidectomy/RET negative/26 LNs were involved with metastasis/elevated calcitonin and CEA/reevaluation with CT scan showed hila, mediastinum, lungs, liver, bones, and kidney were progressively involved/C634R mutation in the RET proto‐oncogene was detected.	Two craniotomies were performed for resection of the large right occipital lesion and another large right parietal lesion. Received adjuvant radiotherapy (60 Gy)/after the relapse of the disease, cabozantinib was initiated; however, 6 months later, spine, bilateral adrenal, and liver were involved. The patient was dead due to hypercortisolism.
Recurrent metastatic medullary thyroid carcinoma: A case of sustained response to prolonged treatment with somatostatin analogues/Cano J. M. et al. [21]	64‐year‐old male	Diagnosis of MTC in 2010/recurrent metastatic disease, diagnosed by elevated CEA and calcitonin in 2012, the patient positive uptake in the right adrenal gland and pancreatic head/a further CT scan revealed metastases in the right adrenal gland, the duodenal bulb, and two pancreatic lesions, which were later confirmed as metastases by endoscopic ultrasound and cytology.	Total thyroidectomy with neck dissection (Stage IVA, pT2pN1bM0, R1) plus adjuvant locoregional radiotherapy after the initial diagnosis in 2010/after recurrence in 2012, Somatuline Autogel was initiated every 28 days, and 11 months later, it declined calcitonin and CEA. A new CT scan showed that the metastatic lesions had disappeared or shrunk in 2016 when a new Octreoscan revealed recurrent disease in the right adrenal gland, a nodule in the right upper pulmonary lobe, and nodal disease in the celiac trunk. CEA and calcitonin were almost remained normal.
Brain metastasis from medullary thyroid carcinoma/Borcek P. et al. [22]	50‐year‐old female	Had been diagnosed with MTC 13 years before presentation/prior developed a cerebellar metastasis which was incidentally discovered/right cerebellar hemispheric mass with contrast enhancement on CT scans/histopathologic exam demonstrated a metastatic tumor composed of nodules and sheets of large tumor cells with abundant cytoplasm/MTC was confirmed in IHC.	By a midline suboccipital incision, the cerebellar tumor was resected, and then tyrosine kinase inhibitor vandetanib RCT was initiated for the patient. The remission of the disease has continued.
Cardiac Metastasis from Medullary Thyroid Cancers with Long‐Term Survival under Vandetanib/Buffet C. et al. [23]	45‐year‐old woman	MTC was diagnosed after total thyroidectomy and neck dissection (11‐mm thyroid tumor with minimal extra‐thyroidal extension and massive lymph node metastases). RET gene analysis was negative/a progressive rise in calcitonin. CEA was seen after 3 years of remission/further assessment showed costal metastases treated with EBRT and surgery 8 years after the initial diagnosis/a rise in calcitonin and CEA led to the diagnosis of liver metastasis and a 30‐mm cardiac mass in the right ventricular apex of MTC metastasis. Two years after the cardiac metastasis diagnosis, the liver metastasis increased in size significantly.	After the detection of cardiac metastasis, radioimmunotherapy with iodine‐131‐labeled anti‐CEA antibodies was performed due to high surgical risk/also, vandetanib (300 mg/day) was administered initially, and the cardiac metastasis remained globally stable after radioimmunotherapy. The liver metastasis, taken as a target lesion, decreased in size until complete response/7 years after vandetanib initiation. The cardiac metastasis decreased slightly on CT. No cardiological metastatic‐related symptom was reported from the diagnosis. Vandetanib was responsible for moderate toxicity: asthenia, increased QTc interval photosensitization with the need for transitory withdrawal, and reduced dosage. Finally, 10 years after its initiation, vandetanib was stopped because of severe functional renal failure. The evaluation at 2.5 years after vandetanib withdrawal showed a stable disease on imaging with stable tumoral marker levels.
Testicular and inguinal lymph node metastases of medullary thyroid cancer: a case report and review of the literature/Appetecchia M. et al. [24]	73‐year‐old male	The patient was diagnosed with MTC 10 years before the current presentation, and in the process of treatment, lumbar vertebrae, lungs, and sacrum metastasis had been diagnosed/the patient presented with a painless nodule in the right testis and a palpable right inguinal lymph node/hypoechoic and inhomogeneous solid mass of 17 mm in size in the upper lobe of the right testis and a 2 cm inguinal LN/a slight increase in two lung nodules in the chest CT scan.	Total thyroidectomy and LN dissection of the neck had been done after the initial diagnosis of MTC in 2002/right radical orchiectomy and excision of the inguinal lymph node were done due to being suspicious of primary testicular cancer metastases, which were identified in IHC morphologically resembling MTC, both in the testis and inguinal LN/calcitonin and CEA levels declined after the surgery/progression of the disease was seen in the lung, bone and inguinal LNs/despite being asymptomatic he was going to start vandetanib at the time of submission of the paper.

Noteworthy, even after the total thyroidectomy, close follow‐up with regular calcitonin measurement should be continued. After this period, any patient with a calcitonin level higher than 100 ng/mL should be thoroughly investigated since this calcitonin level can indicate recurrence, remaining or metastatic MTC [25]. The following steps are determined based on the calcitonin level measured 2–3 months after the surgery, the patient's clinical condition, and the detected oncogenes. For those with MEN2‐positive MTCs, an annual assessment for pheochromocytoma and hyperparathyroidism is highly recommended [5].

The prognosis of MTC patients depends on several factors, such as (1) the stage of the disease, (2) the remaining tumor, (3) age, and (4) histological grade. Even though the survival rate of Grades 1–3 of MTC is 93% in 5 years, this rate for Stage 4 declines to 28% [5, 26]. To determine the patient's prognosis, Fuchs T. et al. concluded that merely histologic features significantly predicted reduced overall survival were the Ki67 proliferative index, mitotic count, and coagulative necrosis. According to the International Medullary Thyroid Carcinoma Grading System (IMTCGS), the Ki67 index < 5% was associated with lower‐grade MTC, while the Ki67 index ≥ 5% was associated with high‐grade MTC [27]. They added these three factors to the age, clinical condition, and MEN2 mutation to predict the survival rate as precisely as possible [28].

The points that should be appreciated in this patient are the concomitant COVID‐19 infection and the invasive metastatic MTC, as well as the susceptibility of patients with malignancies to these viral infections. Moreover, concomitant COVID‐19 infection could also have an impact not only on clinical presentation but also on clinical course and the lack of response to treatment. Although the most common presentation of COVID‐19 is respiratory symptoms, patients might be affected by multiorgan and severe infections that can lead to respiratory distress and be potentially lethal [29]. Furthermore, patients with chronic diseases such as cancers and weak immune systems are more susceptible to severe forms of COVID‐19 [30]. This point should be acknowledged during the treatment planning and admission of patients.

Almost 15%–20% of MTC cases are diagnosed when they are accompanied by distant metastasis; these cases can be severely fatal. Therefore, when encountering unusual presentations in patients with positive MTC history, such as pancytopenia, adrenal insufficiency, abnormal bleeding, and other suggestive signs and symptoms, exceptional locations of the metastasis, such as adrenal and bone marrow, should be kept in mind and ruled out. To make a definite diagnosis or rule out the involvement of these organs, modalities such as bone marrow aspiration, contrast‐enhanced CT scan, or MRI or PET scan might be necessary to be done.

The main strength of this study is the rarity of the metastasis site, which has been reported uncommonly. Unfortunately, the lack of appropriate documentation of the initial presentation and the concomitant COVID‐19 infection has caused a less clear presentation of the initial manifestation. Moreover, the patient died a few days after the presentation and the diagnosis; therefore, expanding follow‐up and elaborating on the optimal approach needed to be sufficiently discussed.

## Author Contributions

**Pouya Ebrahimi:** data curation, supervision, project administration, writing – original draft, writing – review and editing, resources, methodology, conceptualization. **Moloud Payab:** data curation, supervision, resources, project administration, writing – review and editing, writing – original draft, methodology, conceptualization. **Alireza Shariati:** data curation, formal analysis, visualization. **Neda Alipour:** data curation, formal analysis, visualization. **Aysan Nozheh:** investigation, validation, data curation. **Seyed Mohammad Tavangar:** investigation, data curation, validation. **Homa Taheri:** writing – original draft, writing – review and editing, data curation. **Mahbube Ebrahimpur:** data curation, resources, supervision, project administration, writing – review and editing, writing – original draft.

## Ethics Statement

Written informed consent was obtained from the patient's relatives to publish this report under the journal's patient consent policy.

## Conflicts of Interest

The authors declare no conflicts of interest.

## Data Availability

The data that support the findings of this study are available on request from the corresponding author. The data are not publicly available due to privacy or ethical restrictions.
